# Supplementary material for: Early commitment and robust differentiation in colonic crypts
Source: Mol Syst Biol. 2017 Jan 3;13(1):902. doi: 10.15252/msb.20167283 (PMC5293156; doi:10.15252/msb.20167283)
Supplement: Supplementary file 1 — Appendix [file MSB-13-902-s001.pdf]

Appendix

Table of contents

Simulations of stem cell commitment models

1. Modeling cell differentiation in the crypt under early commitment .
2. Late stochastic commitment.
3. Lateral inhibition in the commitment zone as a mechanism for noise buffering .
4. Goblet cell dispersal as a strategy to buffer noise.

Appendix Figures

Appendix Figure S1
Appendix Figure S2
Appendix Figure S3
Appendix Figure S4
Appendix Figure S5
Appendix Figure S6
Appendix Figure S7
Appendix Figure S8

Simulations of stem cell commitment models

Stem cell dynamics in intestinal crypts have been studied using diverse mathematical models, ranging from spatial models through compartment models to non-spatial stochastic models (Johnston *et al*, 2007; Matteis *et al*, 2012; Meineke *et al*, 2001; Mirams *et al*, 2012; Zhao & Michor, 2013; Lopez-Garcia *et al*, 2010; Ritsma *et al*, 2014; Snippert *et al*, 2010; Kozar *et al*, 2013; Vermeulen *et al*, 2013; Buske *et al*, 2011). The simulations we applied in this study were not aimed at achieving a precise description of crypt cellular dynamics but rather to serve as a tool to explore the effect of different processes of proliferation, migration, dispersion and neighbor cell interactions on the variability in stem cell fate.

1. Modeling cell differentiation in the crypt under early commitment

Here we refer to the simulations described in **Fig 1C,E** (red dashed line) and **Fig 3B** in the main text.

Two-dimensional packing

Intestinal crypts are three-dimensional cup-shaped structures, the walls of which are comprised of single-layered epithelial tissue. Cells in the crypt are arranged in a hexagonal configuration, so that six neighbors surround each cell. To simulate dynamics in the crypt, we simplified the system by assuming a two-dimensional flat sheet of cells. All simulations were performed on a hexagonal lattice consisting of 22 rows and 16 columns. Such packing was obtained using Voronoi diagrams, as implemented in Matlab. We imposed continuous boundary conditions as in the real crypt, so that columns 1 and 16 were treated as neighboring columns. Importantly we treat the crypt as a cylinder rather than a cup-shaped structure.

Compartments in the crypt

Recent work based on mathematical modeling of the crypt monoclonalization process converged on an estimate of 6-8 crypt stem cells (Kozar *et al*, 2013; Vermeulen *et al*, 2013; Ritsma *et al*, 2014). We therefore fixed the number of crypt stem cells (SC) in our model at 8 per crypt. Lgr5+ cells populate the bottom 5-6 rows in the crypt, but for simplicity we positioned them in a single row at the lower-most portion of the crypt (**Fig 1C**, green), intermingled between differentiated deep secretory cells (**Fig 1C**, black, (Rothenberg *et al*, 2012), which we assumed to be immotile and fixed to the basal membrane (similarly to the small intestinal Paneth cells). The TA compartment consists of 6 rows of dividing cells immediately above the SC compartment. The remaining upper 15 rows of the crypt consist of differentiated non-dividing cells (the differentiated compartment). At the upper-most part of the differentiated compartment, cells are shed into the lumen.

Cell division throughout the crypt

Cell division at the proliferative portion of the crypt creates mitotic pressure that pushes the cells above towards the lumen. In our simulation setup, each SC has a neighboring SC on each side and three non-SC neighboring cells above. Though the stem cells are not in direct contact with each other, since they are separated by the anchored deep secretory cells, they can interact directly by their ability to replace neighboring SC progenies. This process is similar to the replacement of stem cells that are separated by Paneth cells observed in the small intestine (Ritsma *et al*, 2014; Snippert *et al*, 2010; Lopez-Garcia *et al*, 2010; Vermeulen *et al*, 2013). It leads to the neutral drift dynamics where most labeled clones disappear whereas a few take over the entire crypt, dynamics which we also observed in our short-term lineage tracing (**Appendix Figure S1A**). Thus, SC progenies can be either SCs or not, depending on whether division occurred before the SC was extruded from the SC compartment due to division of neighboring SCs. In the remaining crypt we assumed interaction only between immediate neighbors. Thus, a SC can divide in five directions: sideways and thereby push an adjacent SC outside the SC-niche, or

upwards in three directions ('up', 'up left' or 'up right'). We assumed that division has the same probability of occurring in any direction.

When a SC divides sideways, so that its progeny replaces a neighboring SC, the entire column of the neighboring SC is shifted upwards, so that the former-SC that was just displaced will be situated outside of the SC-niche and therefore irreversibly lose stemness (Walther & Graham, 2014). The upper-most cell in the column that was displaced is shed into the lumen and therefore the total number of cells in the crypt remains fixed. Similarly, if a SC divides upwards in any of the three possible directions, its progeny replaces the non-SC above and a similar shift of the column upwards is implemented. Imposing continuous boundary conditions as in the crypt, cells in column 16 were considered to be in contact with cells in column 1. In these simulations, once a SC divides and a new cell is added to the TA compartment, the new cell is assigned with a secretory or absorptive identity with a respective probability of 25% and 75%, to establish the 1:3 ratio between these lineages. Once allocated to a certain lineage, the cell and all of its future descendants remain committed and do not change their differentiated fate.

Cell division in the TA compartment is carried out in a similar manner, however unlike the SC compartment cells only divide upwards to one of the three directions (with equal probabilities). Another difference is that the TA contains two populations of cells, secretory and absorptive cells, which can differ in their division rates. In simulations shown in **Fig 1B-C** and **1E** (red dashed line), we assumed the same division rates for all cell types (SC, secretory or absorptive). In the differentiated compartment, cells do not divide.

Dwell time in the different simulated compartments

An important feature of the system described is that cells situated in higher rows within the TA compartment have more rows of dividing cells below them. This leads to an acceleration of cells along the crypt axis, and to a respective decreased dwell

time in each row as cells migrate upwards (**Fig 1D**). Dwell time is minimal (and equal) throughout the differentiated compartment, where cells are exposed to the mitotic pressure of the six rows of proliferating cells comprising the TA compartment. In our simulations, while a cell dwells on average for ~4.8 days in the first row of the TA compartment (just above the row of SCs), the dwell time throughout the differentiated compartment decreases to ~0.5 a day.

As explained in the main text, this feature favors early commitment, since the alternative of late commitment would mean rapid extrusion of cells from the crypt shortly after (and perhaps sometimes even before) complete maturation. On the other hand, cell acceleration strongly impinges on the ability to maintain robust proportions of goblet cells between crypts; by the time 8 SCs have divided once (after 2.5 days, see section below), the differentiated compartment will have been fed by 80 cells, since the top row of the TA compartment contains 16 cells, each with an average dwell time of ~0.5 days.

Initial conditions and division-time assignment for newly divided cells

We initiated each simulation by assigning each cell in the TA and differentiated compartments with a random identity (secretory or absorptive). We also initiated all cells in the crypt (except the deep secretory cells) with a random time after which these cells should divide. Division times were chosen randomly from a lognormal distribution (Hawkins *et al*, 2007):

[1] $t^{next\ division} = lognrnd(\mu, \sigma)$

Where lognrnd is the Matlab command for generating random numbers from a lognormal distribution, and:

$m = 60\ hours$ (2.5 days)

$v = ({}^{m}/_{2})^{2}$

$$\mu = \ln\left(\frac{m^2}{\sqrt{v + m^2}}\right)$$

$$\sigma = \sqrt{ln\left(\frac{v}{m^2} + 1\right)}$$

Distribution parameters were chosen so that mean division rate is 2.5 days (**Appendix Figure S1B**) and the minimum division times (dictated by the standard deviation) were no less than ~10 hours – the minimal time for the cell cycle to complete (Quastler & Sherman, 1959). We have chosen 2.5 days since this is the average division rate we obtained from the clonal growth rates (**Appendix Figure S1**). Our results are not sensitive to the precise proliferation rates, and specifically remain unchanged when SCs divide slower than TA cells.

<u>Numeric simulations</u>

After setting the initial conditions, each simulation was solved in discrete time steps. Starting at time $t = 0$, the current time in each iteration is given by $t = i \cdot dt$, where i is the iteration index ($i = 1,2,3 \ldots$) and $dt = 10^{-2}$ hours (~ 0.5 minute). At each time step we checked whether $t > t^{\text{next division}}$ for any given cell, namely whether it was time for that cell to divide. Following division of a cell (SC or non-SC), its two progeny cells were assigned independently with new division times $t^{\text{next division}} + t'$ after which they will each divide again. t' is the time when the division of their mother-cell occurred and $t^{\text{next division}}$ is a value drawn independently for both cells from the lognormal distribution described in Eq. 1. Note that here we assumed no synchronization in divisions, namely cells do not inherit their division rates from their ancestors, nor do they have correlated division rates with their sibling cells; the progeny of each cell is assigned with division rates anew, chosen randomly from the distribution above. Each simulation was run until the random initial cells (initial conditions) were replaced, so that the crypt composition of secretory and absorptive cells only reflected the consequence of the dynamics described.

Estimates of the fraction of SC-traced clones that originate from SCs that have not divided within the SC compartment

In our study we aimed at tracing clones that originate from the very first TA progenitor. Tracing TA progenitors is not feasible with existing lineage tracing mice models, as there is no driver gene specifically expressed only at the bottom TA layer. Tracing clones originating in Lgr5+ SCs should enrich for such clones, however the neutral drift dynamics of SC division can give rise to clones that originate from SCs that have divided within the SC compartment. Indeed, using our lattice simulation we estimated that 50% of the clones include such a division within the SC compartment. We argued that focusing on clones with no SCs in them would enrich for clones that originated in SCs that have not divided within the SC compartment before being extruded. Indeed, when simulating clones in our lattice model we find that only 50% of such clones have divided within the SC compartment before extrusion. Thus focusing on these clones significantly enriches for clonal progenies of a single SC.

Simulation results

Fig 1E and **Fig 3B** (red dashed lines) represent the results of 100 simulations as described above. At the end of each simulation we sampled a patch of cells from the middle of the differentiated compartment and counted the number of secretory and absorptive cells. The patches were continuous and of any random size of 20-100 cells, similar to our experimental measurements (**Table EV3**). Note that here the sample sizes (of 20-100 cells) were larger than the 8 differentiating SCs; while the variability of goblet cells upon SC differentiation, before amplification, can be approximated by the variability of 8 coin tosses with probability 25% (CV of 0.61), the variability between our samples was lower (CV of 0.57, **Fig 7A**), as the patches sampled often included the progenies of more than 8 SCs.

In our clonal analysis, we measured different proliferation rates for the two differentiated cell types (**Fig 6C**), with terminal clone sizes of 3±1 and 5±2.5 cells (median±median absolute deviation) for goblet-cell clones and absorptive clones respectively. To capture this difference in proliferation dynamics we repeated our simulations with a division rate of once every 91 hours for goblet cell clones and once every 48 hours for absorptive clones. The CV of the stochastic commitment simulations were identical when including these differential proliferation rates, demonstrating robustness to proliferation rates (**Appendix Figure S6F**).

2. Late stochastic commitment

In our simulations of late commitment (**Fig 1B,E**, blue dashed line) secretory fate was stochastically assigned to cells with probability 25% as they entered the lowest row of the differentiated compartment. All other simulation properties were identical to the case of early commitment. Late stochastic commitment led to a significantly lower variability compared to the early stochastic commitment; since the average sampled patch was of ~60 cells, the expected CV of goblet proportions can be obtained by dividing the standard deviation of a binomial distribution with parameters $n = 60$ and $p = 0.25$ by its mean. Indeed, our simulations for the late commitment scenario yield a CV of ~0.24, similar to the CV expected.

3. Lateral inhibition in the commitment zone as a mechanism for noise buffering

Here we refer to the simulations described in **Fig 5D,E** in the main text. We simulated 2D packing and cell division as described above for **Fig 1B,C**. We now added to our simulations the commitment zone; at the lower two rows in the TA compartment, cells underwent lateral inhibition (LI) through Delta-Notch signaling (Collier *et al*, 1996; Sprinzak *et al*, 2011). Thus, two adjacent Delta cells that belong to the secretory (goblet) lineage inhibit each other to the point that one of them

differentiates into a Notch cell compatible with the absorptive lineage. The configuration of absorptive and secretory cells was kept throughout the simulation as shown in **Fig 5D**. This layer of LI ensured a tight proportion of 25% secretory cells just above the SC compartment, thereby buffering stochastic noise arising from the differentiating SCs. The LI configuration in our simulations placed goblet cells at even columns. In our simulation geometry even columns were pushed out more rapidly since SCs were positioned in even columns and the quiescent deep secretory cells in odd columns. To counterbalance the resulting higher flux of goblet cells into the differentiated compartment we therefore imposed a slower proliferation rate for goblet cells compared to absorptive cells. To this end, the lognormal distributions in Eq. 1 from which division times were chosen were now centered at different values according to cell identity (SC, secretory or absorptive): For stem cells and for the secretory (Delta) lineage we chose $m^{Delta} = m = 72$ and $v = (^{m}/_{2.5})^2$. For cells from the absorptive (Notch) lineage we chose $m^{Notch} = 47$ and $v^{Notch} = (^{m^{Notch}}/_{3})^2$. Otherwise, the dynamics describing cell differentiation and division were conducted as described above; each SC that exited the SC compartment differentiated and was assigned a division time according to its identity.

Fig 5E presents the results of 100 simulations in which random patches of cells from the differentiated compartment were sampled after the initial conditions were replaced, as described above. **Fig 5D** demonstrates that incorporating the commitment zone reduced the CV dramatically to ~0.26. This effect of reduced variability was insensitive to fluctuations of SC numbers and dynamics (**Appendix Figure S6**).

4. Goblet cell dispersal as a strategy to buffer noise

Here we refer to the simulations described in **Fig 6E,F** in the main text.

Simulating goblet cell dispersal and slower migration

The dispersive goblet cell migration we observed might be a result of several processes, including stronger attachment of goblet cells to the basal membrane, compared to enterocytes, homotypic negative interactions between goblet cells or rather heterotypic attractive adhesion of goblet cells and enterocytes. To capture the dispersion process we implemented the following phenomenological process. We simulated 2D packing, cell division and the commitment zone as described in the section above. To simulate goblet-cell dispersal and slower migration, every time interval of seven hours we identified goblet-cell clusters of two or more adjacent cells, and attempted to break them up by swapping between goblet cells in the cluster and absorptive cells below (thus capturing slower goblet migration). For each goblet cell that had one adjacent goblet neighbor or more, we searched whether it had a neighboring absorptive cell below it. In the case of two or three absorptive lower neighbors, one was chosen randomly. We then attempted to swap between the goblet and absorptive cells and verified that the swap did not result in a new equal or higher neighboring goblet configuration. If swapping indeed resulted in new goblet neighboring, we attempted a swap with a different absorptive lower neighbor if present. In cases where goblet dispersal did not reduce goblet neighboring, swapping was not performed.

To prevent goblet cell accumulation just above the commitment zone, in these simulations the commitment zone was not fixed; after a goblet cell in the upper row of the commitment zone divided, it was swapped with the absorptive cell below, and swapped back again only after its progeny was pushed to a higher position. This swapping did not affect the CV results but reproduced the goblet neighbor profile of **Fig 6B**. Besides dispersion of goblet clusters, to capture the slower migration rate of isolated goblet cells, we attempted to swap goblet cells that did not have goblet neighbors with a lower absorptive cell with some probability (20% every 7 hours), provided it did not result in new cases of neighboring. Indeed, the goblet neighboring profile we obtained was now similar to the one we observed in our experiment (**Appendix Figure S7**).

Simulating synchronization in cell division

In all simulations previously described we assumed no synchronization in division rates, namely division times were chosen for each progeny anew, irrespective of their ancestor's division rate. Upon imposing full synchronization, each SC progeny that was extruded from the SC compartment and differentiated was assigned a division rate as before. Following division in the TA compartment, each dividing cell passed on its division rate to its progeny cells. Thus, all clonal descendants of a differentiated SC were assigned with the same division rates. Synchronization in division is not necessarily absolute; a cell might inherit its division rate to one of its progenies, or to none of them under certain circumstances. As described below, we also accounted for partial synchronization by implementing inheritance with different probabilities; each newly divided cell was assigned a new random division rate with probability p, or inherited its division rate from its progenitor cell with probability 1-p.

Simulation results

In **Fig 6E-F** we sought to test how synchronization in division affects robustness under different degrees of dispersal (mixing). **Fig 6F** presents the CV calculated for different configurations of dispersal/synchrony probabilities. A higher mixing probability results in higher chances for dispersal to occur every seven hours (i.e. if mixing probability is 0.5 there is a chance of 50% for mixing to occur). Similarly, higher synchronization probability results in a higher chance of a dividing cell to pass on its division rate to its progeny cells. The dispersal/mixing process has a direct effect on the mean proportion of goblet cells in the crypt; for a given division rate of goblet cells, a higher dispersal probability leads to an increase in the mean proportion since goblet cells are extruded from the crypt less frequently and dwell longer in the TA compartment where they can divide more. A lower dispersal rate,

on the other hand, leads to the goblet cell depletion. To control for CV increment due to a decreased number of goblet cells, we maintained a mean goblet proportion of 25%±2% in all simulations by normalizing the goblet-cell division rate; for the configuration of no synchronization and 100% mixing, we chose $m^{Delta} = 93hrs$ $m^{SC} = m^{Notch} = 47hrs$. For other configurations of synchronization /mixing probability, we normalized m^{Delta} so that the mean goblet proportion stays in the desired range. Importantly, the increase in CV with decreased mixing and increased synchronization of divisions was insensitive to the precise proliferation parameters.

Fig 6F demonstrates that goblet cell dispersal indeed reduced the CV to the levels we measured experimentally. A significant reduction in the CV was obtained practically irrespective of synchronization levels. At lower levels of goblet cell dispersal on the other hand, our simulations became much more sensitive to synchronization levels; when synchrony was high, a small number of rapidly dividing cells could take over large portions of the crypt, resulting in a high variation in the number of goblet cells between crypts or between epithelial patches within the same crypt (region 1 in **Fig 6E-F**). Dispersive migration alleviates these effects, as it operates to spread the rapidly dividing cells (region 3 in **Fig 6E-F**).

Appendix Figures

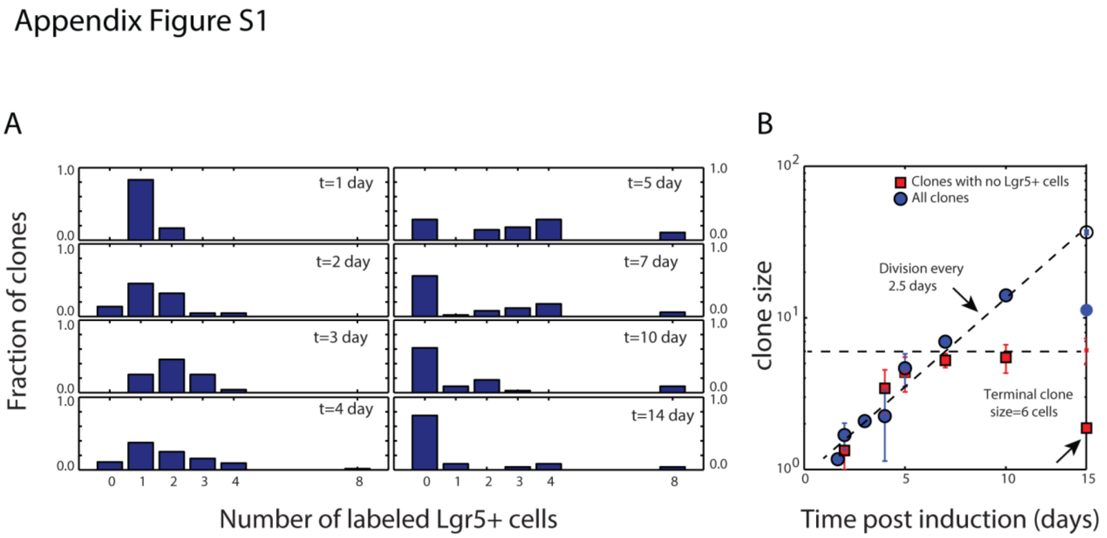

Appendix Figure S1 - Short-term lineage tracing of Lgr5+ progenies enables reconstruction of colonic crypt proliferative parameters.

A Patterns of short-term lineage tracing of Lgr5-cell progenies follow a neutral drift dynamic. Shown are the probability distributions of the numbers of Lgr5+ cells in clones at sequential days following tamoxifen induction. Crypts with mixed SC compartments (including both labeled and unlabeled SC) become exceedingly rare as time proceeds.

B Proliferation rate in the colonic crypt is on average once every 2.5 days. Blue circles denote clones that have at least one Lgr5+ cell, red squares denote clones that have no Lgr5+ cells, representing, with high probability clonal progenies of an Lgr5+ cell that has been extruded from the SC compartment. Average terminal clone size is 6.1±1.2 (median±median absolute deviation 4.5±3). See also **Table EV1**. N=251 clones from 12 mice.

Appendix Figure S2

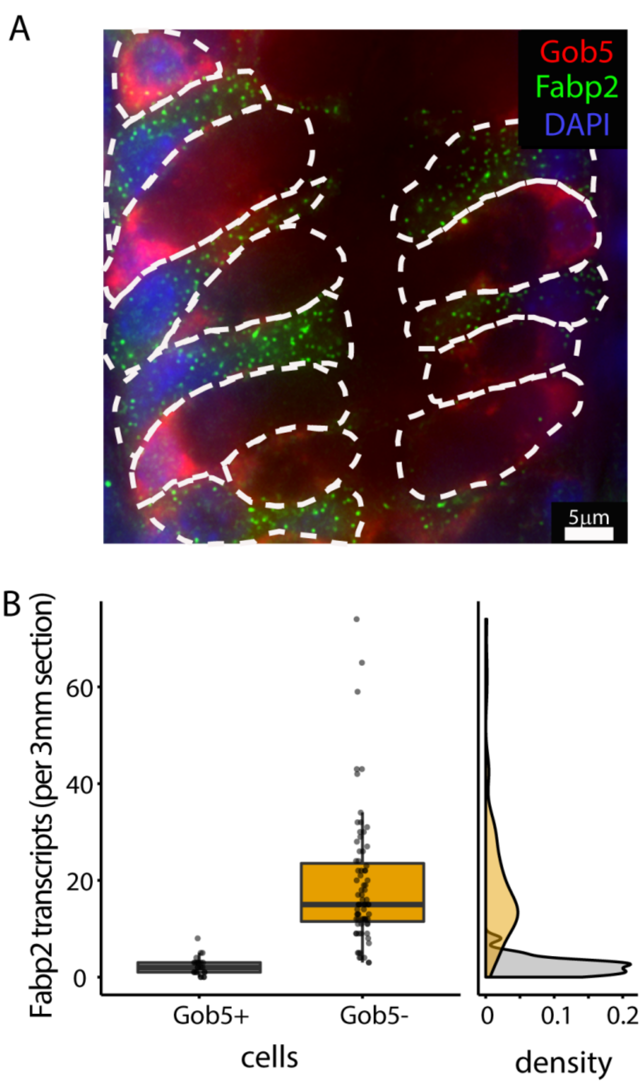

Appendix Figure S2 – Gob5 and Fabp2 are mutually exclusive.

A Differentiation markers for goblet cells and enterocytes respectively. Shown is a mid-crypt region. Green dots are single mRNA of Fabp2, red dots are mRNA of Gob5. Since Gob5 expression is high at these higher crypt positions, individual dots cannot be discerned. Dashed white lines mark cell borders.

B The majority of cells in the colonic crypts are either Gob5+ or Fabp2-high. Cells expressing Gob5 contain low levels of Fabp2, whereas more than 93% of cells which are Gob5 negative express high levels of Fabp2. Left – box plots of cells at the

differentiated compartment (100 cells from the differentiated compartment, 2 mice). Right – density plots of cells according to their Fabp2 expression level. The majority of cells in the colonic crypts are either Gob5 positive or Fabp2-high. The remaining 5% of the cells express Fabp2 at basal levels, which was set as the Fabp2 expression level of the 90th percentile Gob5+ cells. These could be other cell types such as enteroendocrine cells.

Appendix Figure S3

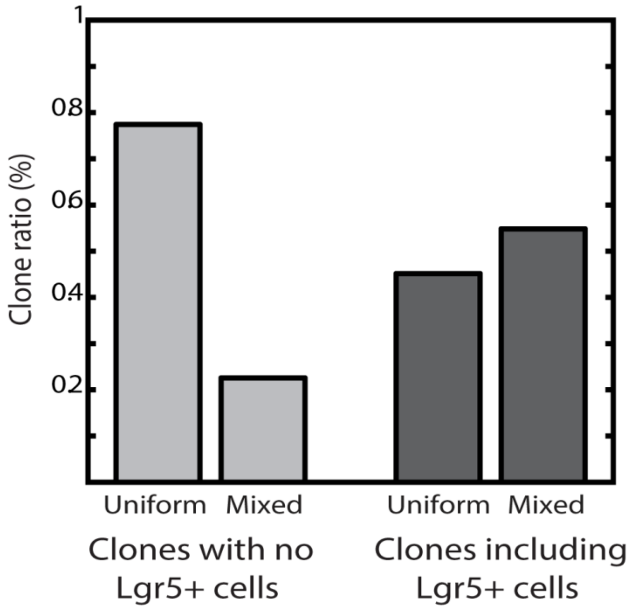

Appendix Figure S3. Clones from Lgr5-CreERT2-Confetti mice that do not contain Lgr5+ cells (light gray, N=119 clones) are predominantly uniform in their fate, whereas clones which contain at least one Lgr5+ cell (dark gray, N=62 clones) are of mixed fate. Measurements are from eight mice in the colon. Data presented in **Table EV2**.

Appendix Figure S4

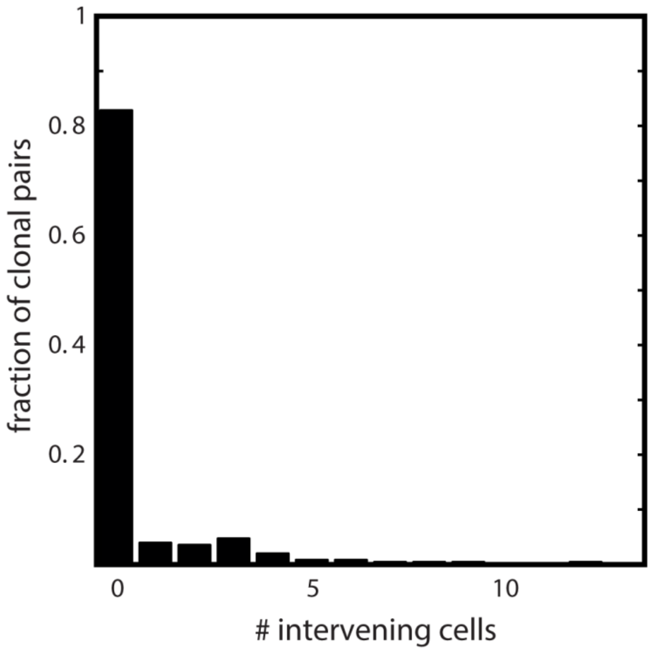

Appendix Figure S4 - Clonal cells are often adjacent to each other. Shown are the distances between closest cells within a clone, where distance is defined as number of non-clonal cells intermingled in between pairs of clonal cells. 82% of the clonal pairs have distance 0, hence are in direct contact. Quantification based on 55 clones positioned in the bottom 10 rows of the crypts. n=256 pairs, 8 mice.

Appendix Figure S5

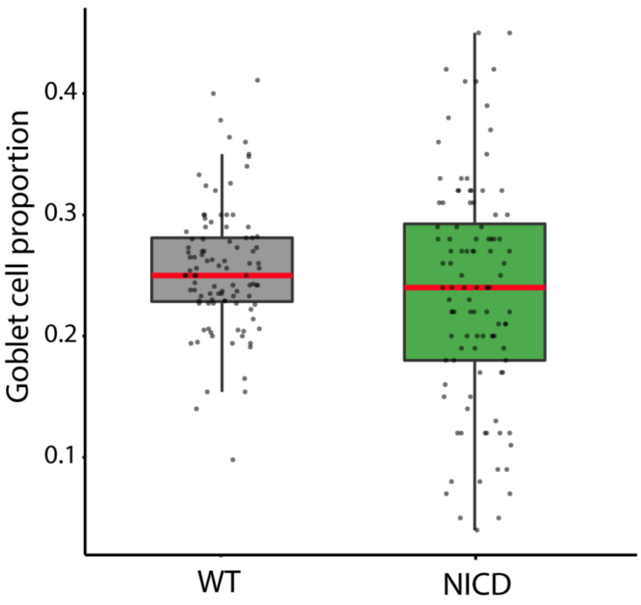

Appendix Figure S5. Perturbation of Notch signaling increases variability of goblet cells proportions. The percentage of goblet cells was calculated for 100 crypts in WT mice and 104 crypts in NICD mice. While maintaining the similar median (red lines, mean=25.6% and 23.7% for WT and NICD respectively), NICD crypts have a wider range of goblet cells proportions among different crypts (CV=0.39 in NICD vs. CV=0.21 in WT).

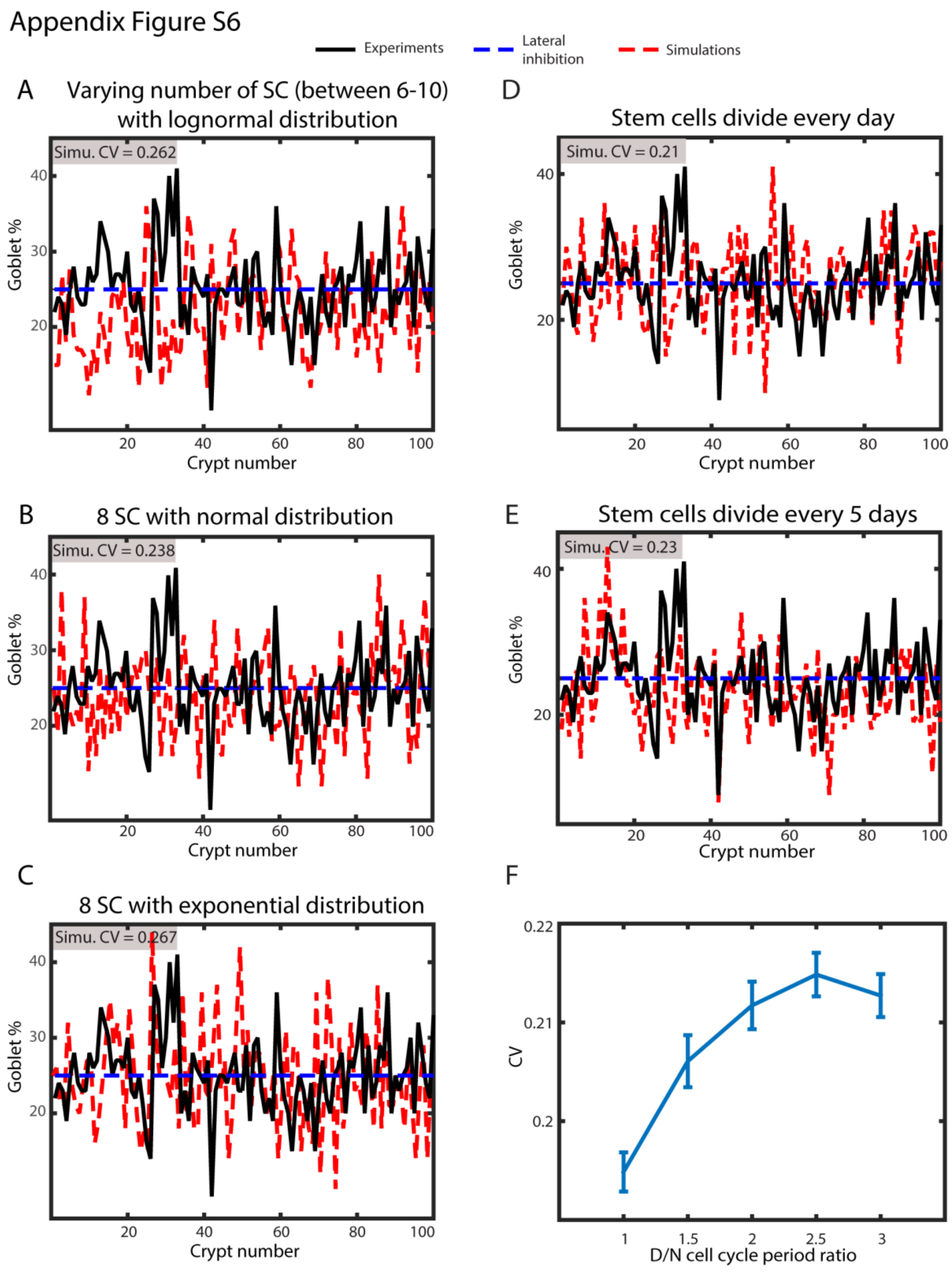

Appendix Figure S6 - Lateral inhibition in the commitment zone confers robustness of goblet cell proportions to fluctuations of SC numbers and dynamics.

A Goblet proportions in 100 simulated crypts (dashed red line), each crypt contained a random number of stem cells, ranging from 6 to 10. Division times were randomly assigned from a lognormal distribution, with mean division time of 72h±28.8h. Division times for Notch and Delta cells were drawn from a lognormal distribution with mean division time of 47h ± 15.66h and 72±28.8h, respectively. Plotted in black in all panels **A-E** is the experimental measurement of goblet proportion in 100 crypts. Dashed blue line in panels **A-E** marks the expected goblet proportion under complete LI (25%).

B Goblet proportions in 100 simulated crypts (dashed red line), in which the division rate of the cells was drawn from a normal distribution. For SCs and goblet cells, mean division time was 72h±11h, for Notch cells, mean division time 47±7hr.

C Goblet proportions in 100 simulated crypts (dashed red line), in which the division rate of the cells was drawn from an exponential distribution. Means of the exponential distributions are equal to those of the normal distributions.

D Goblet proportions in 100 simulated crypts (dashed red line), in which stem cell divide every 24±9.6h. Notch and Delta divisions were drawn as in **A**.

E Goblet proportions in 100 simulated crypts (dashed red line), in which stem cell divide once every 120±61.53h. Notch and Delta divisions were drawn as in **A**.

F Variability in goblet cell proportions is robust to different ratios in cell cycle periods of Delta and Notch cells. Notch cells divide every 47h ± 15.66h. Delta cells divide every 47±15.66, 70.5±28.2, 94±42.72, 117.5±58.75 and 141±74.21h. SCs divide every 72±28.8h. For each division rate configuration, the CV of 100 simulations was calculated 50 times. Shown are the mean CV and standard errors. Since slower Delta division rates were not compensated for by migration (as in main **Figure 6F**), they resulted in a lower mean and therefore a higher CV.

Appendix Figure S7

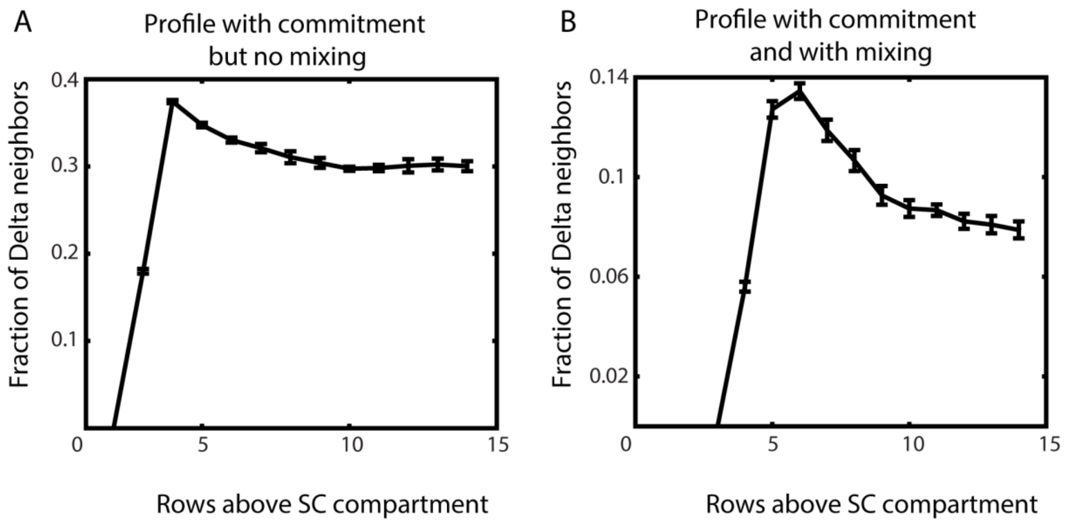

Appendix Figure S7 - **Simulated profile of goblet-cell neighboring as a function of crypt height.**

A Profile in a model that includes lateral inhibition in the commitment zone. The number of goblet neighbors increases sharply in the TA compartment and later saturates in the differentiated compartment.

B Profile in a model that includes lateral inhibition in the commitment zone as well as goblet cell dispersion. In the TA compartment, the number of goblet neighbors increases more moderately compared to **A**. However unlike **A,** the number of goblet neighbors decreases in the differentiated compartment where no division occurs.

Appendix Figure S8

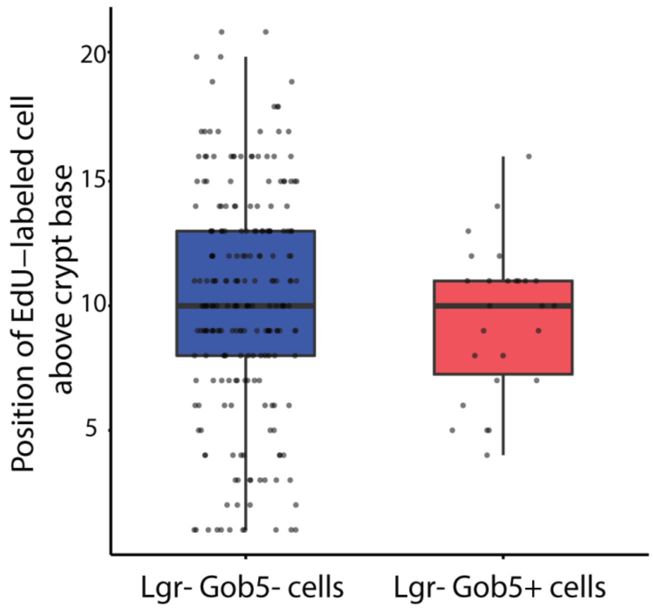

Appendix Figure S8 – Position of dividing goblet cells is indistinguishable from position of dividing enterocytes.

While much less frequent than enterocytes (26 EdU-labeled Goblet cells and 219 EdU-labeled enterocytes), positions of replicating goblet cells are not significantly different than positions of replicating enterocytes. N=2 mice. Mice were injected EdU and sacrificed 1.5h later. EdU detection and smFISH for Gob5, Ki67 and Lgr5 was done in parallel to identify the cycling cells, their position and their type.

Appendix References

Buske P, Galle J, Barker N, Aust G, Clevers H & Loeffler M (2011) A Comprehensive Model of the Spatio-Temporal Stem Cell and Tissue Organisation in the Intestinal Crypt. *PLoS Comput Biol* **7:** e1001045

Collier JR, Monk NA, Maini PK & Lewis JH (1996) Pattern formation by lateral inhibition with feedback: a mathematical model of delta-notch intercellular signalling. *J. Theor. Biol.* **183:** 429–446

Hawkins ED, Turner ML, Dowling MR, Gend C van & Hodgkin PD (2007) A model of immune regulation as a consequence of randomized lymphocyte division and death times. *Proc. Natl. Acad. Sci.* **104:** 5032–5037

Johnston MD, Edwards CM, Bodmer WF, Maini PK & Chapman SJ (2007) Mathematical modeling of cell population dynamics in the colonic crypt and in colorectal cancer. *Proc. Natl. Acad. Sci. U. S. A.* **104:** 4008–4013

Kozar S, Morrissey E, Nicholson AM, van der Heijden M, Zecchini HI, Kemp R, Tavaré S, Vermeulen L & Winton DJ (2013) Continuous Clonal Labeling Reveals Small Numbers of Functional Stem Cells in Intestinal Crypts and Adenomas. *Cell Stem Cell* **13:** 626–633

Lopez-Garcia C, Klein AM, Simons BD & Winton DJ (2010) Intestinal Stem Cell Replacement Follows a Pattern of Neutral Drift. *Science* **330:** 822–825

Matteis GD, Graudenzi A & Antoniotti M (2012) A review of spatial computational models for multi-cellular systems, with regard to intestinal crypts and colorectal cancer development. *J. Math. Biol.* **66:** 1409–1462

Meineke FA, Potten CS & Loeffler M (2001) Cell migration and organization in the intestinal crypt using a lattice-free model. *Cell Prolif.* **34:** 253–266

Mirams GR, Fletcher AG, Maini PK & Byrne HM (2012) A theoretical investigation of the effect of proliferation and adhesion on monoclonal conversion in the colonic crypt. *J. Theor. Biol.* **312:** 143–156

Quastler H & Sherman FG (1959) Cell population kinetics in the intestinal epithelium of the mouse. *Exp. Cell Res.* **17:** 420–438

Ritsma L, Ellenbroek SIJ, Zomer A, Snippert HJ, de Sauvage FJ, Simons BD, Clevers H & van Rheenen J (2014) Intestinal crypt homeostasis revealed at single-stem-cell level by in vivo live imaging. *Nature* **507:** 362–365

Rothenberg ME, Nusse Y, Kalisky T, Lee JJ, Dalerba P, Scheeren F, Lobo N, Kulkarni S, Sim S, Qian D, Beachy PA, Pasricha PJ, Quake SR & Clarke MF (2012) Identification of a cKit(+) colonic crypt base secretory cell that supports Lgr5(+) stem cells in mice. *Gastroenterology* **142:** 1195–1205.e6

Snippert HJ, van der Flier LG, Sato T, van Es JH, van den Born M, Kroon-Veenboer C, Barker N, Klein AM, van Rheenen J, Simons BD & Clevers H (2010) Intestinal Crypt Homeostasis Results from Neutral Competition between Symmetrically Dividing Lgr5 Stem Cells. *Cell* **143:** 134–144

Sprinzak D, Lakhanpal A, LeBon L, Garcia-Ojalvo J & Elowitz MB (2011) Mutual Inactivation of Notch Receptors and Ligands Facilitates Developmental Patterning. *PLoS Comput Biol* **7:** e1002069

Vermeulen L, Morrissey E, Heijden M van der, Nicholson AM, Sottoriva A, Buczacki S, Kemp R, Tavaré S & Winton DJ (2013) Defining Stem Cell Dynamics in Models of Intestinal Tumor Initiation. *Science* **342:** 995–998

Walther V & Graham TA (2014) Location, location, location! The reality of life for an intestinal stem cell in the crypt. *J. Pathol.* **234:** 1–4

Zhao R & Michor F (2013) Patterns of Proliferative Activity in the Colonic Crypt Determine Crypt Stability and Rates of Somatic Evolution. *PLoS Comput Biol* **9:** e1003082
